# Controllable sites and high-capacity immobilization of uranium in Nd_2_Zr_2_O_7_ pyrochlore

**DOI:** 10.1107/S1600577521012558

**Published:** 2022-01-01

**Authors:** Jian Sun, Jing Zhou, Zhiwei Hu, Ting-Shan Chan, Renduo Liu, Haisheng Yu, Linjuan Zhang, Jian-Qiang Wang

**Affiliations:** a Key Laboratory of Interfacial Physics and Technology, Shanghai Institute of Applied Physics, Chinese Academy of Sciences, Shanghai 201800, People’s Republic of China; b University of Chinese Academy of Sciences, Beijing 100049, People’s Republic of China; c Max Planck Institute for Chemical Physics of Solids, Nöthnitzer Strasse 40, 01187 Dresden, Germany; d National Synchrotron Radiation Research Center, 101 Hsin-Ann Road, Hsinchu 30076, Taiwan

**Keywords:** molten salt method, actinide immobilization, Nd_2_Zr_2_O_7_, X-ray absorption spectroscopy

## Abstract

As potential nuclear waste host matrices, two series of uranium-doped Nd_2_Zr_2_O_7_ nanoparticles were successfully synthesized using an optimized molten salt method in an air atmosphere. Our combined X-ray diffraction, Raman and X-ray absorption fine-structure (XAFS) spectroscopy studies reveal that uranium ions can precisely substitute the Nd site to form an Nd_2–*x*_U_*x*_Zr_2_O_7+δ_ (0 ≤ *x* ≤ 0.2) system and the Zr site to form an Nd_2_Zr_2–*y*_U_*y*_O_7+δ_ (0 ≤ *y* ≤ 0.4) system without any impurity phase. With increasing U concentration, there is a phase transition from pyrochlore (*Fd* 3 *m*) to defect fluorite (*Fm* 3 *m*) structures in both series of U-doped Nd_2_Zr_2_O_7_. The XAFS analysis indicates that uranium exists in the form of high-valent U^6+^ in all samples. To balance the extra charge for substituting Nd^3+^ or Zr^4+^ by U^6+^, additional oxygen is introduced accompanied by a large structural distortion; however, the Nd_2_Zr_1.6_U_0.4_O_7+δ_ sample with high U loading (20 mol%) still maintains a regular fluorite structure, indicating the good solubility of the Nd_2_Zr_2_O_7_ host for uranium. This study is, to the best of our knowledge, the first systematic study on U-incorporated Nd_2_Zr_2_O_7_ synthesized via the molten salt method and provides convincing evidence for the feasibility of accurately immobilizing U at specific sites.

## Abstract 2

The first systematic study on U-incorporated Nd_2_Zr_2_O_7_ synthesized via a molten salt method for potential actinide immobilization is presented.

## 1. Introduction

The disposal of actinide-rich nuclear waste is a key issue for the development of sustainable nuclear energy (Ewing, 2011 ▸; Abdou *et al.*, 2018 ▸; Finkeldei *et al.*, 2020 ▸; Liu *et al.*, 2021 ▸). The search for high-capacity, low-cost and radiation-tolerant materials that can be used as host materials for nuclear wastes has been a major research interest for a long time. Pyrochlore oxides have received considerable attention in immobilizing actinides owing to their desirable physical-chemical properties, including their high thermal stability, high chemical durability, and strong resistance to radiation damage (Abdou *et al.*, 2018 ▸; Fuentes *et al.*, 2018 ▸; Finkeldei *et al.*, 2020 ▸). Pyrochlore, ideally *A*_2_ *B*_2_O_7_, can accommodate a variety of chemical compositions, in which typically a trivalent cation occupies the *A* sites coordinated by eight oxygen atoms and tetravalent cation occupies the *B* sites coordinated by six oxygen atoms (Marlton *et al.*, 2021 ▸; Talanov & Talanov, 2021 ▸). Among them, zirconate pyrochlore is the most important class of host materials due to its remarkable resistance to amorphization under ion beam irradiation (Lu, Shu, Chen *et al.*, 2018 ▸; Lu, Shu, Shao *et al.*, 2018 ▸; Lu *et al.*, 2019 ▸; Shu *et al.*, 2017 ▸). Previous studies have demonstrated that actinides can be effectively incorporated into the zirconate pyrochlore matrix at both the *A* and *B* sites (Blackburn *et al.*, 2021 ▸; Jafar *et al.*, 2015 ▸; Lu *et al.*, 2017 ▸; Shu *et al.*, 2016 ▸; Wang *et al.*, 2021 ▸), but many impurities, such as ZrO_2_, U_3_O_8_, ThO_2_ and other complex uranium oxides, were introduced at high actinide concentrations, and the stability of the pyrochlore was reduced. Moreover, the immobilization capacity, local structure and valence state of the incorporated actinides (*i.e.* their speciation) have not been fully explored in previous reports. Realizing the rational manipulation of the phase structure and substituting sites in zirconate pyrochlore, as well as developing low-cost and sustainable synthetic methods, remains a tremendous challenge, which has practical significance in the application of immobilizing actinides.

The conventional method for the preparation of zirconate pyrochlore is a solid-state reaction, which normally results in inhomogeneity and impurities in the final phases (Shu *et al.*, 2016 ▸). To improve the chemical homogeneity and phase accuracy of the system, several wet-chemistry routes have been employed for the preparation of these zirconium-based pyrochlore materials, which involves the mixing of reactant precursors and fast mass transport at the molecular level, resulting in better compositional homogeneity and controlled stoichiometric ratio of the obtained samples. However, subsequent sintering is generally carried out at temperatures above 1400°C for more than 48 h, which is of high cost and not suitable for large-scale waste disposal (Lu, Shu, Chen *et al.*, 2018 ▸). Principally, molten salt synthesis is a potential methodology that can lower the reaction temperature due to faster mass transport. In previous work (Mao *et al.*, 2009 ▸; Gupta & Mao, 2021 ▸), La_2_Zr_2_O_7_ pyrochlore ceramic was synthesized via the molten salt method by heating mixtures at 650°C for 6 h. However, until now, only limited work has been reported on actinide-incorporated pyrochlore via the molten salt method (Abdou *et al.*, 2018 ▸, 2019 ▸; Wang *et al.*, 2020 ▸). Neodymium zirconate has also been proposed as a potential ceramic matrix for the immobilization of nuclear waste; therefore, in this work, we chose Nd_2_Zr_2_O_7_ as a suitable host material for studying uranium immobilization via a molten salt process. We successfully synthesized two series of Nd_2_Zr_2_O_7_ (NZO) nanoparticles doped with different U concentrations by an optimized molten salt method and achieved precise control of doping at either Nd or Zr sites to form Nd_2–*x*_U_*x*_Zr_2_O_7+δ_ or Nd_2_Zr_2–*y*_U_*y*_O_7+δ_, respectively. X-ray diffraction, Raman and transmission electron microscopy were used to characterize the crystalline structure, phase transition and morphology in U-doped NZO nanoparticles. XAFS was used to obtain detailed information on the oxidation state and local environments of metal ions.

## 2. Experimental methods and characterization

### 2.1. Reagents

The reagents used in this experiment include Nd(NO_3_)_3_·6H_2_O (99.9%), ZrO(NO_3_)_2_·2H_2_O (99.5%), UO_2_(NO_3_)_2_·6H_2_O, NH_4_OH (28%), NaNO_3_ (99.9%) and KNO_3_ (99.9%). All reagents are used as received without further purification.

### 2.2. Material synthesis

All samples were synthesized by an innovative molten salt method (Mao *et al.*, 2009 ▸). In the first step, the single-source composite precursor material was synthesized by the coprecipitation method. The original reactants contained Nd(NO_3_)_3_·6H_2_O, ZrO(NO_3_)_2_·2H_2_O and UO_2_(NO_3_)_2_·6H_2_O. The reactants were measured stoichiometrically as (5−α) mmol Nd(NO_3_)_3_·6H_2_O, 5 mmol ZrO(NO_3_)_2_·2H_2_O and α mmol UO_2_(NO_3_)_2_·6H_2_O corresponding to *A* site uranium doping, and 5 mmol Nd(NO_3_)_3_·6H_2_O, (5−β) mmol ZrO(NO_3_)_2_·2H_2_O and β mmol UO_2_(NO_3_)_2_·6H_2_O corresponding to *B* site uranium doping. All reagents were dissolved in 200 ml deionized water and stirred at 400 rev min^−1^. Then, 200 ml of dilute ammonia [NH_4_OH:H_2_O = 1:9 (*v*/*v*)] solution was dropped into the solution, forming a flocculent and exhibiting stratification. The stabilized precipitate was centrifuged out and dried in an oven at 80°C for 24 h to obtain a composite homogeneous composite precursor (2−*x*)Nd(OH)_3_·2ZrO(OH)_2_·*x*UO_2_(OH)_2_·*n*H_2_O and 2Nd(OH)_3_·(2−*y*)ZrO(OH)_2_·*y*UO_2_(OH)_2_·*n*H_2_O]. In the second step, NZO and uranium-doped NZO were synthesized by the molten salt method. 0.35 g of the precursor, 2.55 g of NaNO_3_ and 3.03 g of KNO_3_ were placed into an agate mortar and ground for 30 minutes. The fine mixture was then placed in a corundum crucible and annealed at 750°C for 6 h with a heating rate of 5°C min^−1^. After natural cooling, the mixture was soaked and washed with deionized water five times to remove the residual molten salt. Finally, the sample was dried at 80°C for 12 h to obtain the product. By controlling the stoichiometric ratio of the initial reactants, NZO and U-substituted NZO materials were obtained, including compounds NZO, Nd_1.9_U_0.1_Zr_2_O_7+δ_ (*A*−0.1), Nd_1.8_U_0.2_Zr_2_O_7+δ_ (*A*−0.2), Nd_1.6_U_0.4_Zr_2_O_7+δ_ (*A* − 0.4), Nd_2_Zr_1.9_U_0.1_O_7+δ_ (*B*−0.1), Nd_2_Zr_1.8_U_0.2_O_7+δ_ (*B*−0.2) and Nd_2_Zr_1.6_U_0.4_O_7+δ_ (*B*−0.4).

### 2.3. Characterization

The crystal structure was analysed by powder X-ray diffraction spectroscopy (XRD) on a Bruker D8 advanced AXS diffractometer with Cu *K*α (λ = 1.54178 Å) irradiation and micro-Raman spectroscopy on a Horiba LabRAM HR Evolution with laser wavelength of 633 nm. The lattice parameters were obtained by Rietveld refinement using *GSAS II* software (version 4300) (Toby & Von Dreele, 2013 ▸). The ideal pyrochlore structure model was used, assuming the 8a and 48f sites fully occupied. The morphology was characterized by scanning electron microscopy (SEM; Zeiss LEO 1530VP) and transmission electron microscopy (TEM; FEI Tecnai G2 F20 S-TWIN). The X-ray absorption spectra experiments were performed at BL14W1 in the Shanghai Synchrotron Radiation Facility (SSRF) and BL16A1 in the National Synchrotron Radiation Research Center (NSRRC). The storage ring of SSRF was operated at 3.5 GeV with a maximum current of 260 mA. Using a Si (111) double-crystal monochromator, the data collection was carried out in transmission mode for the Zr *K*-edge with an ionization chamber and in fluorescence mode for the U *L*_3_-edge with a Lytle detector. The storage ring of NSRRC was operated at 1.5 GeV with a maximum current of 360 mA. Using a double-crystal monochromator, the sample was placed in an argon atmosphere, and the data collection was carried out in fluorescence mode for the Nd *L*_3_-edge with a Lytle detector. All data were collected at ambient temperature. The XAFS data were analysed using the program *Demeter* (Ravel & Newville, 2005 ▸). For extended X-ray absorption fine-structure (EXAFS) data fitting of Zr-*K*, the Fourier transform window of the *k* space was of the sine type in the range 3.0–12.0 Å, and the sine type of the *R* space was in the range 1.0–4.0 Å. The amplitude reduction factor 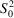 was fixed as 0.75 during EXAFS fitting, based on the fitting result for the known structure of Zr foil. For EXAFS data fitting of U-*L*_3_, the Fourier transform window for *k* space was of the sine type in the range 2.0–8.5 Å and sine type for *R* space in the range 1.0–5.0 Å. The amplitude reduction factor 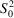 was set to 0.9 (Zhang *et al.*, 2018 ▸).

## 3. Results and discussion

### 3.1. X-ray diffraction

**Figure 1:**
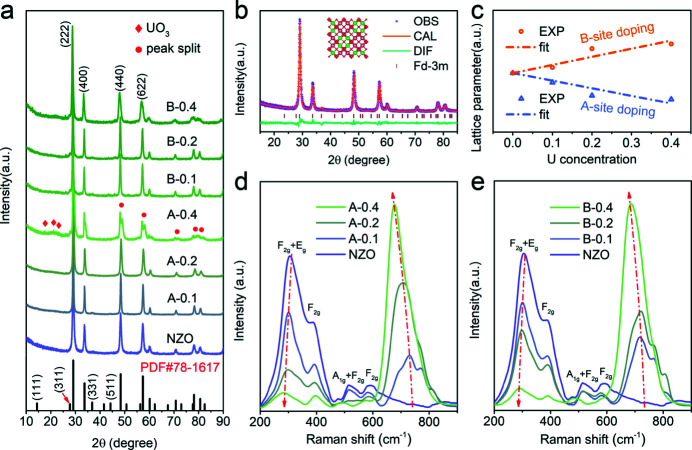
Structural characterization. (*a*) XRD profiles of Nd_2_Zr_2_O_7_, Nd_2–*x*_U_*x*_Zr_2_O_7+δ_ (*A*−0.1, *A*−0.2, *A*−0.4) and Nd_2_Zr_2–*y*_U_*y*_O_7+δ_ (*B*−0.1, *B*−0.2, *B*−0.4). (*b*) Rietveld refinement of XRD profile for Nd_2_Zr_2_O_7_ (NZO). (*c*) Evolution of the fitted lattice parameters with the U concentrations. Raman spectra of (*d*) Nd_2–*x*_U_*x*_Zr_2_O_7+δ_ samples and (*e*) Nd_2_Zr_2–*y*_U_*y*_O_7+δ_ samples.

The crystal structures of pure Nd_2_Zr_2_O_7_ (NZO) and U-doped NZO materials synthesized by the optimized molten salt method were characterized by powder X-ray diffraction. As displayed in Fig. 1 ▸(*a*), the diffraction peaks of the as-prepared NZO sample are in good agreement with the reflections of a typical pyrochlore structure (space group 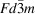 ; PDF: 78-1617). However, the superlattice peaks of (111), (311), (331) and (511) for the NZO sample are relatively weak, indicating relatively poor crystallinity and the possible existence of defective fluorite structures (space group: 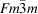 ) (Hagiwara *et al.*, 2019 ▸). Rietveld refinement of the XRD pattern demonstrates the purity of the NZO sample with a pyrochlore structure [Fig. 1 ▸(*b*)], and the parameters are summarized in Table S1 of the supporting information.

The U-doped NZO samples have a similar XRD pattern as the pyrochlore structure, supporting the introduction of uranium into *A* or *B* sites to generate Nd_2–*x*_U_*x*_Zr_2_O_7+δ_ or Nd_2_Zr_2–*y*_U_*y*_O_7+δ_, respectively (Gupta *et al.*, 2016 ▸). Compared with that of NZO, the diffraction patterns of all U-doped NZO samples show increasing broadening with increasing uranium substitution level. Such broadening XRD patterns could be ascribed to an increase in lattice distortions induced by the different effective ionic radii of the U (0.73 Å for six-fold coordination and 0.86 Å for eight-fold coordination), Nd (1.11 Å for eight-fold coordination) and Zr (0.72 Å for six-fold coordination) ions (Wang *et al.*, 2021 ▸; Ziolkowski, 1985 ▸). This may even lead to possible phase transitions in the sample with high U content. In the Nd_1.6_U_0.4_Zr_2_O_7+δ_ (*A*−0.4) sample, as marked in Fig. 1 ▸(*a*), second-phase UO_3_ (PDF: 45-0856) was observed, and the diffraction peaks in the high-angle area split, indicating the existence of other oxides, such as Nd_0.5_Zr_0.5_O_1.75_ (PDF: 78-1289) or Nd_0.2_Zr_0.8_O_1.9_ (PDF: 78-1301). As shown in Fig. 1 ▸(*c*), we clearly observed an opposite trend in the lattice parameters, which shrink in Nd_2–*x*_U_*x*_Zr_2_O_7+δ_ samples but expand in Nd_2_Zr_2–*y*_U_*y*_O_7+δ_ samples with increasing uranium content. Considering that the effective radius of U^6+^ ions is between that value of Nd^3+^ and Zr^4+^ ions, the shrinkage or expansion of lattice parameters is easy to understand, which supports that uranium ions can precisely replace either Nd or Zr sites in NZO hosts, as expected. Quantitative structural parameters are summarized in Table S1, and the *x*–O_48*f*_ positional parameters in Nd_2–*x*_U_*x*_Zr_2_O_7+δ_ and Nd_2_Zr_2–*y*_U_*y*_O_7+δ_ increase regularly with increasing uranium content (except for *A*−0.4), implying that the crystal structure undergoes a phase transition from the pyrochlore phase to the defect fluorite phase due to the distortion of the ZrO_6_ octahedron (Wang *et al.*, 2018 ▸). However, *A*−0.4 does not conform to the rule due to the appearance of an impurity phase and phase separation, indicating that the introduction of uranium at the *A* site by the molten salt method has reached the limit. Clearly, it is much easier for uranium ions to accommodate at Zr sites than at Nd sites. For this type of substitution, no significant phase impurities can be observed. The transition from ordered to dis­ordered pyrochlore phase and defect fluorite phase cannot be well distinguished by XRD alone. However, these results can be supplemented by Raman spectroscopy.

### 3.2. Raman spectroscopy

The sensitivity of Raman spectroscopy to oxygen displacements makes it widely used to distinguish pyrochlore and fluorite structures (Mączka *et al.*, 2009 ▸; Pokhrel *et al.*, 2016 ▸; Sanjuán *et al.*, 2011 ▸). In fluorite ( 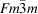 ), only one Raman active mode is expected. In a perfect pyrochlore ( 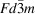 ), there are six Raman active vibrational modes: *T* = *A*_1*g*_ + *E*_*g*_ + 4*F*_2*g*_ (Turner *et al.*, 2017 ▸), in which *A*_1*g*_ belongs to O–*B*–O bending vibration (∼520 cm^−1^), *E*_*g*_ is mainly attributed to the O–B–O bending vibration and *B*–O stretching vibration (∼300 cm^−1^), and *F*_2*g*_ is the O–*B*–O_6_ octahedral vibration and O–*A*–O_48*f*_ bending vibration (∼300 cm^−1^, 400 cm^−1^, 500 cm^−1^, 600 cm^−1^) (Niu *et al.*, 2019 ▸; Park *et al.*, 2018 ▸; Zhang *et al.*, 2010 ▸). As shown in Figs. 1 ▸(*d*) and 1(*e*), the Raman spectra of NZO were in good agreement with the peaks of the pyrochlore phase. With increasing U doping amount, the intensity of the Raman spectra in Nd_2–*x*_U_*x*_Zr_2_O_7+δ_ and Nd_2_Zr_2–*y*_U_*y*_O_7+δ_ begins to weaken and shifts to low frequency, which is consistent with Maya Abdou’s result (Abdou *et al.*, 2018 ▸). These changes reflect the increase in the structural disorder degree, especially for anions (Zhang *et al.*, 2014 ▸). In particular, the peak intensity decreases to its lowest at *A*−0.4 and *B*−0.4, suggesting that the pyrochlore phase was gradually transformed into a disordered fluorite phase. In addition, *A*−0.1 and *B*−0.1 exhibit three additional Raman active sites (∼720 cm^−1^, 770 cm^−1^, 820 cm^−1^), which may be related to the distorted *B*–O_6_ octahedron induced by the incorporated U cations (Garg *et al.*, 2008 ▸; Tang *et al.*, 2020 ▸). Similar modes were found in other uranium compounds (Gregg *et al.*, 2013 ▸; Manara & Renker, 2003 ▸; Palacios & Taylor, 2000 ▸; Abdou *et al.*, 2018 ▸; Tang *et al.*, 2020 ▸), such as U_3_O_8_, UO_3_ and UO_2_^2+^ clusters. In U_3_O_8_ and UO_3_, 700 cm^−1^ is attributed to the stretching of U-O, and 760 cm^−1^ is related to the symmetric stretching of O=U=O, despite the slightly lower frequency offset. Through semiquantitative analysis of the Raman peak intensity at 700–800 cm^−1^, we can obtain the corresponding relationship between the doping level of uranium and the structural transformation. The intensity of the peaks at 700–800 cm^−1^ increases significantly with increasing U substitution, which indicates that uranium ions can be successfully introduced into the parent phase and form a stable conformation. On the other hand, compared with that of *A*−0.2, the three Raman active sites (∼720 cm^−1^, 770 cm^−1^, 820 cm^−1^) are well maintained in the *B*−0.2 sample, implying that the structural incorporation of uranium ions at the *B* site is better than that at the *A* site. When the U concentration reaches 20 mol% of the substituted site, both *A*−0.4 and *B*−0.4 show significant structural evolution, which is consistent with the XRD results (Gupta *et al.*, 2016 ▸).

### 3.3. Morphological characterization

**Figure 2:**
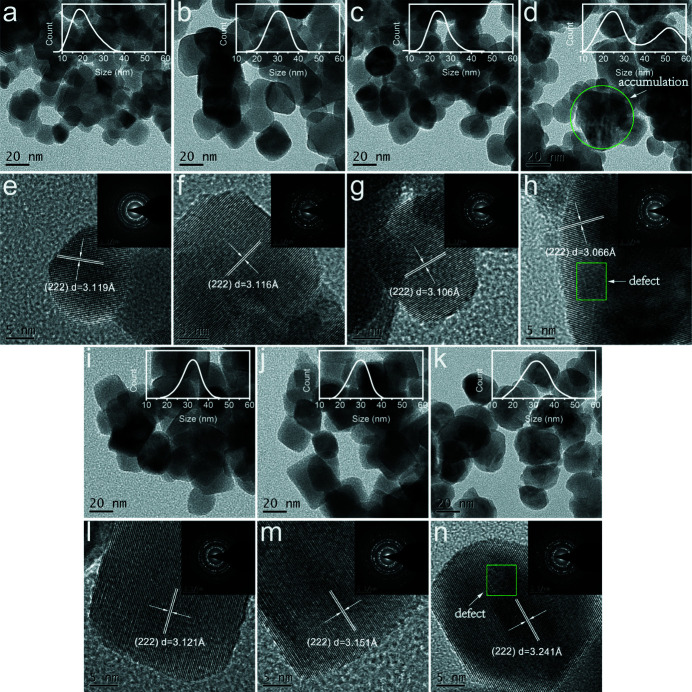
TEM images and HR-TEM results of U-doped NZO samples. Panels (*a*, *e*) refer to the NZO samples; (*b*, *f*), (*c*, *g*), (*d*, *h*) refer to the *A*−0.1, *A*−0.2 and *A*−0.4 samples, respectively; and (*i*, *l*), (*j*, *m*) and (*k*, *n*) refer to the *B*−0.1, *B*−0.2 and *B*−0.4 samples, respectively; additionally, the illustration shows the corresponding SAED result.

The morphology of all samples was characterized by SEM and TEM. The elemental distributions (Figure S2) and molar ratios (Table S2) of all samples were detected by SEM-EDS, which indicated good elemental uniformity in the parent material. As shown in Fig. 2 ▸(*a*), the NZO sample has a polyhedral structure with dimensions of ∼20 nm. This good uniformity is attributed to the fast mass transfer of the reaction components at the atomic scale, which benefits from the optimized molten salt method (Dargaud, Cormier *et al.*, 2010 ▸; Liu *et al.*, 2013 ▸).

Compared with the morphology of the NZO parent phase, the characteristics of the U-doped NZO samples show obvious changes with increasing U concentration. The particle sizes of *A*−0.1 and *B*−0.1 are significantly larger than that of NZO. Such differences reflect that the incorporation of uranium affects the initial growth mode, and a higher content of U may further lead to disordered structures and phase transitions, as confirmed by XRD and Raman results. The crystal grains of *A*−0.2 evolve into a spherical shape, while *B*−0.2 still maintains a uniform particle size distribution with a cubic structure. When the uranium content continues to increase, the particles in *A*−0.4 begin to aggregate and grow into irregular masses, while the particles in *B*−0.4 have a good dispersion despite evolving into irregular shapes, supporting that U doping at *A* sites is much more different. Importantly, the inset in Fig. 2 ▸(*d*) shows a bimodal size distribution, indicating a kind of phase separation in *A*−0.4, consistent with the XRD result. Well defined lattice fringes were detected by high-resolution transmission electron microscopy (HRTEM), and the lattice fringe spacing corresponding to the (222) planes of Nd_2–*x*_U_*x*_Zr_2_O_7+δ_ and Nd_2_Zr_2–*y*_U_*y*_O_7+δ_ have opposite change trends, which is consistent with the XRD results. The inset selected-area electron diffraction (SAED) patterns of all samples show clear polycrystalline diffraction rings, corresponding to the three strong peaks of the pyrochlore or defect fluorite phase. In addition, obvious defects can be observed in the HRTEM for the *A*−0.4 and *B*−0.4 samples [Figs. 2 ▸(*h*), 2(*n*)].

### 3.4. Local electronic and crystal structure

**Figure 3:**
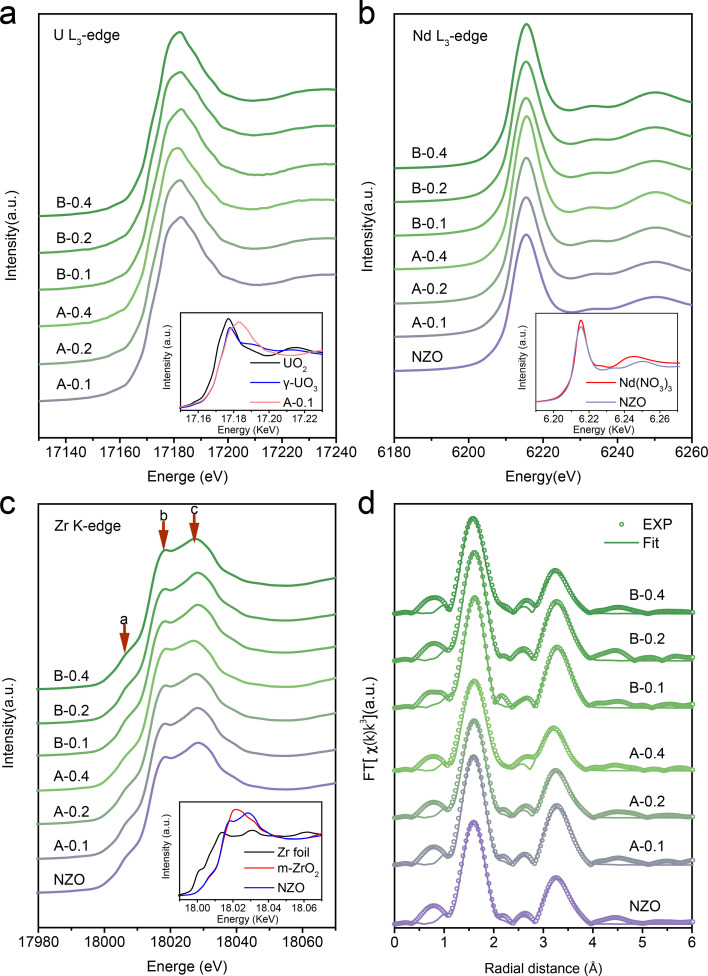
XAFS data of the NZO and U-doped NZO samples. (*a*) U-*L*_3_, (*b*) Nd-*L*_3_ and (*c*) Zr *K*-edge XANES spectra of U-doped NZO samples, (*d*) Fourier transform (FT) of Zr *K*-edge EXAFS data and their corresponding fits in *R* space.

X-ray absorption fine structure (XAFS) is highly sensitive to the local electronic and crystal structures and therefore has been widely used in actinide compounds (Sun *et al.*, 2019 ▸; Zhang *et al.*, 2018 ▸; Zhang, Su *et al.*, 2016 ▸; Zhang, Zhou *et al.*, 2016 ▸; Hu *et al.*, 1998 ▸). Fig. 3 ▸(*a*) shows the X-ray absorption near-edge structure (XANES) spectra of the U *L*_3_-edge for all U-doped NZO samples, together with spectra of UO_2_ and γ-UO_3_ as a U^4+^ reference and a U^6+^ reference, respectively. The energy position of U *L*_3_-edge XANES for U-doped NZO samples is basically the same as that of the γ-UO_3_ standard, indicating a U^6+^ state in all samples. However, to achieve charge balance, the introduction of high-valent U for substituting Nd^3+^ or Zr^4+^ sites should induce a large number of cation vacancies or additional oxygen, along with higher structural disorder (Abdou *et al.*, 2018 ▸; Finkeldei *et al.*, 2020 ▸). It is important to note that the XANES spectral features can be affected by the valence state, local symmetry and structural disorder. Previous works reported that the energy position of spectral features is affected by structural disorder with shifts of approximately ±0.5–1.0 eV (Conradson *et al.*, 2003 ▸, 2004 ▸). The high structural disorder around high-valent U may explain the wide main peak of U *L*_3_-edge XANES and the reason that no significant differences were observed between the Nd_2–*x*_U_*x*_Zr_2_O_7+δ_ and Nd_2_Zr_2–*y*_U_*y*_O_7+δ_ samples in Fig. 3 ▸(*a*). To detect the bonding between uranium and oxygen ligands in U-doped NZO, we performed an extended X-ray absorption fine-structure (EXAFS) analysis of the U *L*_3_-edge by using Fourier transform (FT). Figure S3 shows the *k* ^3^-weighted χ(*k*) signal in the U-doped NZO sample and its corresponding U *L*_3_-edge FT. The broad peak at ∼1.5 Å in the *R* space arising from the U–O coordination shell indicates that there is a high oxygen disorder around the U site. Table S3 shows that this disorder (σ^2^) reaches its highest value at *A*−0.4 and *B*−0.4. At the same time, the coordination number decreases as the uranium concentration increases. It is worth noting that, for the U–O path, Nd_2–*x*_U_*x*_Zr_2_O_7+δ_ has a shorter bond length than Nd_2_Zr_2–*y*_U_*y*_O_7+δ_. This difference implies that the molten salt method realizes the doping of uranium at the *A* and *B* sites of the NZO matrix material and produces a slight difference in the oxygen coordination of uranium.

To assess the structural changes of Nd and Zr affected by dopant U ions in U-doped NZO samples, Nd-*L*_3_ and Zr-*K* XANES measurements were also performed. The Nd *L*_3_-edge XANES spectra of all samples exhibit a similar shape, as shown in Fig. 3 ▸(*b*), and the white line energy position is very close to that of the Nd(NO_3_)_3_ reference, demonstrating the 3+ valence state of the Nd ions. In the case of an increase in the valence by one, the white line maximum would shift by 7–8 eV to higher energies (Lissner *et al.*, 1994 ▸; Hu *et al.*, 1997 ▸). In a typical pyrochlore, Nd coordinates with eight oxygens to form a hexahedral structure, and it contains two different crystallographic oxygen sites: O_8*b*_ and O_48*f*_. Among them, O_48*f*_ has special properties concerning the adjustment of oxygen disorder and structural distortion in pyrochlore (Hagiwara *et al.*, 2019 ▸; Martin *et al.*, 2009 ▸). Previous studies pointed out that the introduction of high-valent ions will cause the 8*a* oxygen vacancies to be partially filled and cause the anion disorder of Gd_2_Zr_*x*_Ti_2–*x*_O_7+δ_ pyrochlore (Nästren *et al.*, 2009 ▸). In the Nd *L*_3_-edge XANES, the strong white line originates from a dipole-allowed transition from core level 2*p* to 5*d*, and the spectral profile is related to the unoccupied Nd 5*d* states (Rao *et al.*, 1983 ▸). As observed in Figure S4, the intensity of the white line at the Nd *L*_3_-edge XANES increases gradually with increasing uranium concentration. This can be mainly assigned to the strong covalent mixing of broad U 6*d* with O 2*p* competing with Nd 5*d*–O 2*p* mixing. Furthermore, U doping leads to an increase in the symmetry of the Nd_2_Zr_2_O_7_ system and, in turn, a narrower 5*d* band. Fig. 3 ▸(*c*) shows the Zr *K*-edge XANES spectra of the NZO and U-doped NZO samples, which can be characterized by an obvious pre-edge structure (labelled by *a*) and a main peak split into two well resolved features (labelled by *b* and *c*). Peaks *a* and *b*/*c* correspond to the electronic transition from the 1*s* to 4*d* orbital and the dipole-allowed transition from the 1*s* to 5*p* orbital, respectively (Komyoji *et al.*, 1992 ▸; Popov *et al.*, 2019 ▸). These features of all samples are close to those in the pyrochlore phase rather than in the defect-fluorite phase (Nandi *et al.*, 2020 ▸). In Zr *K*-edge XANES, the pre-edge feature a for all samples is significant, which indicates tetrahedral distortion of the ZrO_6_ coordination polyhedron (Dargaud, Calas *et al.*, 2010 ▸). The *c*/*b* intensity ratios of all samples are similar to that of pyrochlore-type Gd_2_Zr_2_O_7_ with six-fold coordinated Zr^4+^. The ratio would rather deviate for Zr^4+δ^ (NZO) compounds with seven- and eight-fold coordination (Komyoji *et al.*, 1992 ▸; Galoisy *et al.*, 1999 ▸; Li *et al.*, 1993 ▸). Deeper knowledge of the local structure around Zr can be obtained from the quantitative analysis of EXAFS spectra. As shown in Fig. 3 ▸(*d*), all samples show two main coordination peaks, in which the first peak corresponds to six O_48*f*_ atoms at approximately 2.2 Å, and the second peak corresponds to six Zr ions and six Nd ions. The quantitative results are shown in Table S4.

The Zr *K*-edge EXAFS spectra are sensitive to changes in the local environment with increasing uranium content in the Nd_2–*x*_U_*x*_Zr_2_O_7+δ_ and Nd_2_Zr_2–*y*_U_*y*_O_7+δ_ samples. A noticeable change in the coordination number (CN) is observed for Zr–O_48*f*_ bonds during uranium doping. Compared with that in NZO, the CN values of Zr–O increased slightly in the *A*−0.1 and *B*−0.1 samples, which may be caused by the introduction of anions to maintain charge balance and preferentially fill the unoccupied 8*a* site in the pyrochlore structure (Hess *et al.*, 2002 ▸; Nästren *et al.*, 2009 ▸). Since Zr is located at the 16*c* site (adjacent to the 8*a* site), the addition of Zr–O CN has little effect on the polyhedral structure of Zr–O_48*f*_. However, the CN of Zr–O decreased significantly when keeping increasing the U content in Nd_2–*x*_U_*x*_Zr_2_O_7+δ_ and Nd_2_Zr_2–*y*_U_*y*_O_7+δ_. In this case, more uranium is introduced into pyrochlore, which causes the Zr–O octahedron to be distorted, and the structure has more oxygen disorder characteristics by the movement of O_48*f*_ and the formation of anionic Frenkel defects (Popov *et al.*, 2019 ▸). In particular, this disorder is maximized at the highest doping concentration, which is consistent with the extremely low characteristic peak of pyrochlore in the Raman results. Through conventional Zr *K*-edge EXAFS analysis, the pyrochlore and defect fluorite phases cannot be accurately distinguished. For the coordination of Zr–Zr and Zr–Nd, CN decreases continuously with increasing uranium amount in the Nd_2–*x*_U_*x*_Zr_2_O_7+δ_ and Nd_2_Zr_2–*y*_U_*y*_O_7+δ_ systems, and the bond lengths of Zr–Zr and Zr–La increase regularly. This indicates that the movement of pyrochlore cation sites and the deformation of the pyrochlore lattice lead to the formation of a disordered system.

## 4. Conclusion

In this work, we systematically studied the uranium solubility, crystalline phase, valence state and structural evolution in a Nd_2_Zr_2_O_7_ pyrochlore synthesized by a modified molten salt method. According to the XRD results, uranium ions can be precisely confined at either the Nd or Zr sites in Nd_2_Zr_2_O_7,_ and the solubility can reach up to a 20 mol% substitution at the Zr sites and a 10 mol% substitution at the Nd sites without any impurity phase. The Raman analysis shows that there is a phase transition from ordered pyrochlore to defective fluorite with increasing U concentration. The TEM images show that all U-doped Nd_2_Zr_2_O_7_ samples have good crystallinity and dispersity except for Nd_1.6_U_0.4_Zr_2_O_7+δ_. The U *L*_3_-edge XANES spectra indicate that uranium exists in the form of high-valent U^6+^ in all samples. To balance the extra charge from substituting Nd^3+^ or Zr^4+^ by high-valent U^6+^, additional oxygen is introduced along with a large structural distortion, as revealed by the XAFS analysis. This systematic work sheds light on understanding actinide immobilization, which will accelerate the development of advanced technology in nuclear waste storage materials.

## Supplementary Material

Figures S1 to S4; Tables S1 to S4. DOI: 10.1107/S1600577521012558/ok5057sup1.pdf
