# Supplementary material for: Controllable sites and high-capacity immobilization of uranium in Nd2Zr2O7 pyrochlore
Source: J Synchrotron Radiat. 2022 Jan 1;29(Pt 1):37–44. doi: 10.1107/S1600577521012558 (PMC8733979; doi:10.1107/S1600577521012558)
Supplement: Supplementary file 1 [file s-29-00037-sup1.pdf]

JOURNAL OF SYNCHROTRON RADIATION

Volume 29 (2022)

Supporting information for article:

Controllable Sites and High-Capacity Immobilization of Uranium in Nd2Zr2O7 Pyrochlore

Jian Sun, Jing Zhou, Zhiwei Hu, Ting-Shan Chan, Renduo Liu, Haisheng Yu, Linjuan Zhang and Jian-Qiang Wang

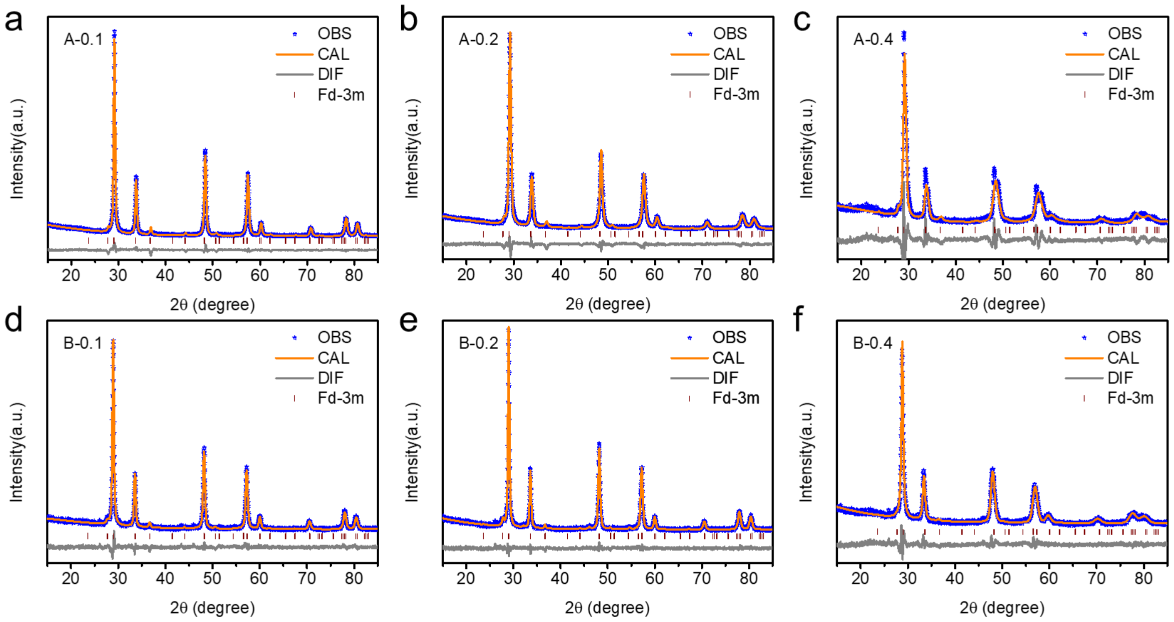

Figure S1 Rietveld refinement results of XRD patterns for $Nd_{2-x}U_xZr_2O_{7+\delta}$ and $Nd_2Zr_{2-y}U_yO_{7+\delta}$ samples.

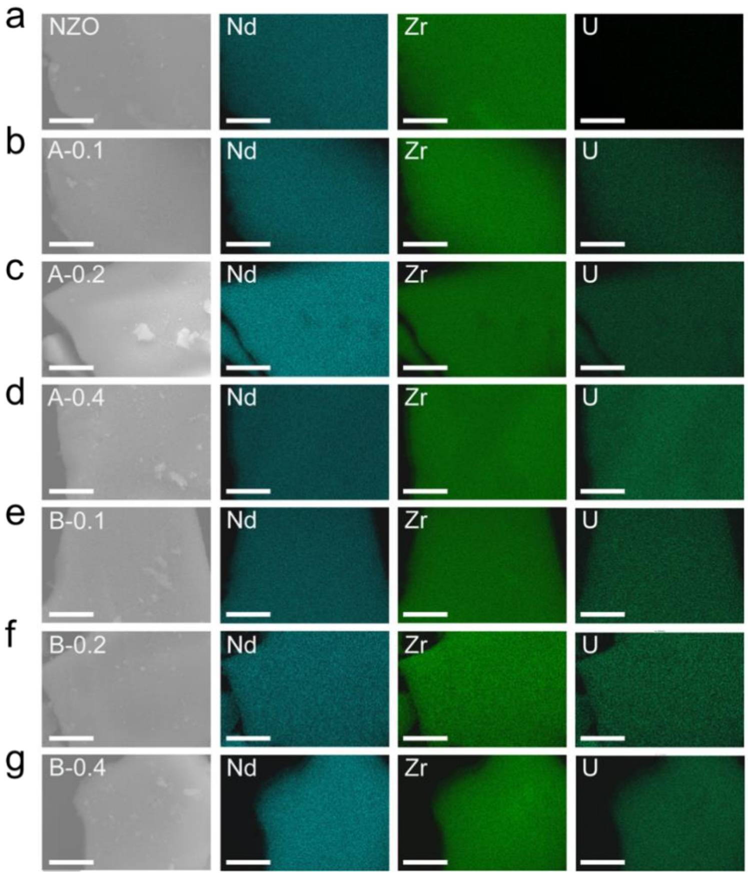

Figure S2 The SEM-EDS results of all samples. All scale bar correspond to 2 μm.

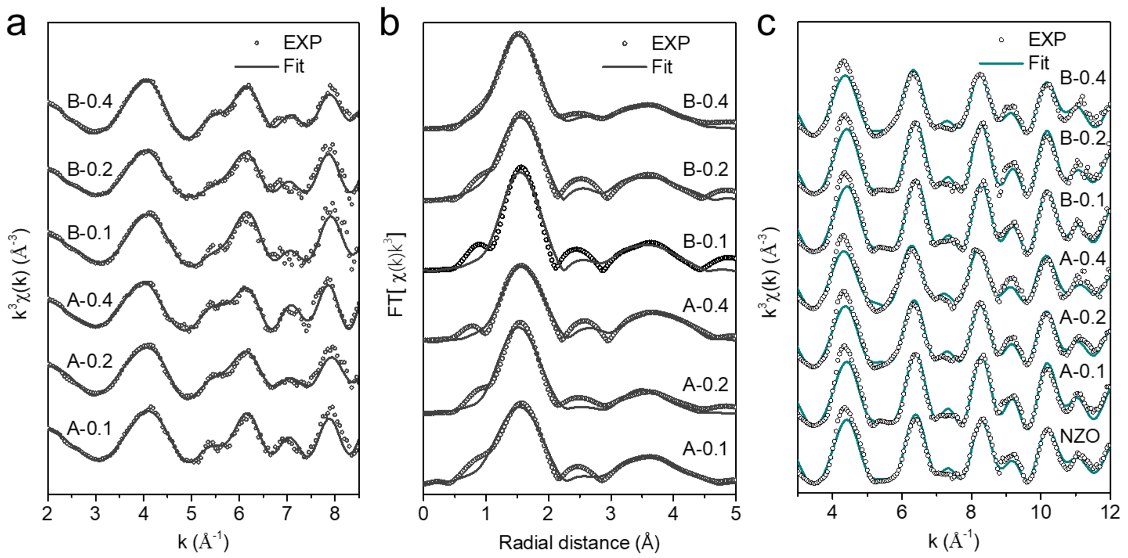

Figure S3 XAFS data of the NZO and U-doped NZO samples. (a) U L_3-edge EXAFS data and their corresponding fits in k space, (b) Fourier Transforms (FT) of U L_3-edge EXAFS data and their corresponding fits in R space and (c) Zr K-edge EXAFS data and their corresponding fits in k space.

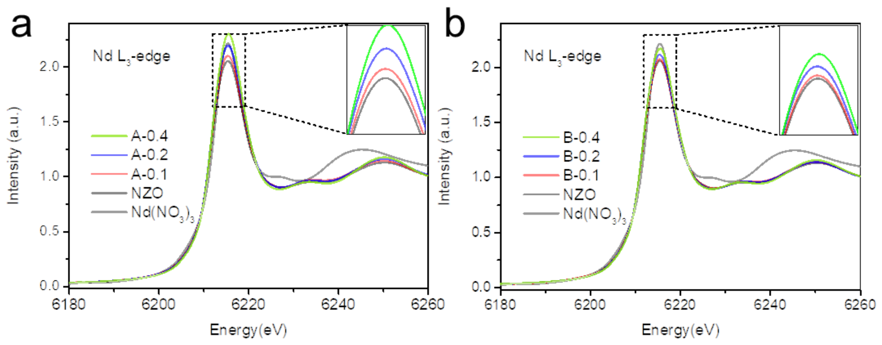

Figure S4 Nd L_3-edge XANES of all samples.

Table S1 Refined structural parameters for all samples.

Sample	a (Å)	Bond distance (Å)			Uiso				x-O_{48f}	Rwp	GOF
		Nd-O_{8b}	Nd-O_{48f}	Zr-O_{48f}	Nd	Zr	O_{8b}	O_{48f}			
NZO	10.663(1)	2.309(1)	2.435(1)	2.195(1)	0.0166(1)	0.0166(1)	0.03	0.03	0.335(1)	13.46	1.45
A-0.1	10.653(1)	2.306(1)	2.450(1)	2.179(1)	0.0160(1)	0.0160(1)	0.03	0.0310(1)	0.339(1)	8.16	1.75
A-0.2	10.639(1)	2.303(1)	2.411(1)	2.205(1)	0.0156(1)	0.0156(1)	0.03	0.0396(1)	0.352(1)	8.21	1.95
A-0.4	10.635(1)	2.306(1)	2.334(1)	2.279(1)	0.0140(1)	0.0140(1)	0.03	0.0420(1)	0.350(1)	14.69	2.55
B-0.1	10.669(1)	2.310(1)	2.393(1)	2.232(1)	0.0169(1)	0.0169(1)	0.03	0.0358(1)	0.338(1)	7.53	1.29
B-0.2	10.689(1)	2.314(1)	2.397(1)	2.237(1)	0.0175(1)	0.0175(1)	0.03	0.0427(1)	0.348(1)	7.12	1.18
B-0.4	10.694(1)	2.316(1)	2.393(1)	2.302(1)	0.0162(1)	0.0162(1)	0.03	0.0424(1)	0.354(1)	9.13	1.47

Table S2 The SEM-EDS results of all samples.

Proportion of atoms	Nd	Zr	U
NZO	2.25	2	0
A-0.1	1.97	2	0.12
A-0.2	1.82	2	0.21
A-0.4	1.56	2	0.39
B-0.1	2	1.89	0.12
B-0.2	2	1.75	0.21
B-0.4	2	1.61	0.44

Table S3 U L_3-edge EXAFS fitting results for all samples.

Sample	Shell	CN	ΔE_0 (eV)	σ^2 (Å^2)	R (Å)	R factor
A-0.1	O1.1	6.4 (6)	4.5 (5)	0.0170 (5)	2.11 (1)	0.02
	Nd1.1	6	10	0.0055 (4)	3.83 (1)	
	Zr1.1	6	10	0.0134 (5)	3.68 (1)	
A-0.2	O1.1	6.0 (3)	5.8 (5)	0.0173 (2)	2.14 (1)	0.03
	Nd1.1	6	10	0.0046 (6)	3.81 (1)	
	Zr1.1	6	10	0.0124 (5)	3.65 (1)	
A-0.4	O1.1	5.6 (3)	6.5 (4)	0.0186 (4)	2.18 (1)	0.02
	Nd1.1	6	10	0.0064 (3)	3.85 (1)	
	Zr1.1	6	10	0.0112 (7)	3.70 (1)	
B-0.1	O1.1	6.3 (9)	4.5 (6)	0.0101 (6)	2.09 (1)	0.03
	Nd1.1	6	10	0.0063 (6)	3.82 (1)	
	Zr1.1	6	10	0.0136 (5)	3.67 (1)	
B-0.2	O1.1	5.8 (5)	3.0 (3)	0.0139 (4)	2.08 (1)	0.03

	Nd1.1	6	10	0.0080 (4)	3.85 (1)	
	Zr1.1	6	10	0.0129 (7)	3.69 (1)	
B-0.4	O1.1	5.7 (4)	4.6 (4)	0.0156 (3)	2.13 (1)	0.01
	Nd1.1	6	10	0.0084 (2)	3.80 (1)	
	Zr1.1	6	10	0.0172 (2)	3.64 (1)	

Table S4 Zr K-edge EXAFS fitting results for all samples.

Sample	Shell	CN	ΔE_0 (eV)	σ^2 (Å^2)	R (Å)	R factor
NZO	O1.1	6	4.5 (2)	0.0048 (3)	2.21 (1)	0.02
	Zr1.1	6	-10	0.0119 (5)	3.81 (1)	
	Nd1.1	6	-10	0.0119 (5)	3.81 (1)	
A-0.1	O1.1	6.1 (3)	4.7 (5)	0.0039 (3)	2.22 (1)	0.02
	Nd1.1	5.9 (5)	-10	0.0098 (4)	3.80 (1)	
	Zr1.1	5.9 (5)	-10	0.0098 (4)	3.80 (1)	
A-0.2	O1.1	5.8 (3)	4.1 (3)	0.0049 (4)	2.23 (1)	0.02
	Nd1.1	5.5 (6)	-10	0.0111 (5)	3.83 (1)	
	Zr1.1	5.5 (6)	-10	0.0111 (5)	3.83 (1)	
A-0.4	O1.1	5.5 (5)	5.8 (4)	0.0058 (6)	2.30 (1)	0.05
	Nd1.1	5.1 (8)	-10	0.0113 (10)	3.90 (1)	
	Zr1.1	5.1 (8)	-10	0.0113 (10)	3.90 (1)	
B-0.1	O1.1	6.2 (2)	4.0 (5)	0.0045 (4)	2.20 (1)	0.02
	Nd1.1	6.0 (5)	-10	0.0106 (5)	3.80 (1)	
	Zr1.1	6.0 (5)	-10	0.0106 (5)	3.80 (1)	
B-0.2	O1.1	6.1 (4)	4.2 (3)	0.0046 (3)	2.21 (1)	0.02
	Nd1.1	5.9 (6)	-10	0.0103 (5)	3.81 (1)	
	Zr1.1	5.9 (6)	-10	0.0103 (5)	3.81 (1)	
B-0.4	O1.1	5.6 (4)	6.8 (3)	0.0053 (6)	2.27 (1)	0.03
	Nd1.1	5.0 (7)	-10	0.0111 (9)	3.85 (1)	
	Zr1.1	5.0 (7)	-10	0.0111 (9)	3.85 (1)	
